# Multicenter surveillance of antifungal susceptibility of clinical *Aspergillus* isolates to conventional and novel antifungal agents in Taiwan, 2021–2023

**DOI:** 10.1128/spectrum.02659-25

**Published:** 2026-04-21

**Authors:** Ming-I Hsieh, Pui-Ching Choi, Wan-Lin Wu, Yu-Fu Lin, Chi-Jung Wu

**Affiliations:** 1 National Institute of Infectious Diseases and Vaccinology, National Health Research Institutes, Tainan, Taiwan; 2 Division of Infectious Diseases, Department of Internal Medicine, National Cheng Kung University Hospital, College of Medicine, National Cheng Kung University 34912, Tainan, Taiwan; Mayo Foundation for Medical Education and Research, Rochester, Minnesota, USA

**Keywords:** amphotericin B, antifungal resistance, *Aspergillus*, azole, echinocandin, ibrexafungerp, manogepix, orolofim, rezafungin, Taiwan

## Abstract

Periodic surveillance of antifungal susceptibility among *Aspergillus* isolates is essential to guide effective treatment. This multicenter study analyzed 550 clinical *Aspergillus* isolates collected in Taiwan from 2021 to 2023, primarily from the respiratory tract (69.6%) and ear (12.9%). Calmodulin-based sequencing identified 24 species across six *Aspergillus* sections: *Flavi* (28.7%), *Fumigati* (28.5%), *Nigri* (21.6%), *Terrei* (15.8%), *Nidulantes* (5.1%), and *Circumdati* (0.2%). The major sections were represented by *Aspergillus fumigatus* (99.4%), *Aspergillus flavus* (91.8%), and *Aspergillus terreus* (100%), while section *Nigri* comprised nine species, with *Aspergillus welwitschiae* (57.1%) predominant. Using Clinical Laboratory Standards Institute (CLSI) M38-A3 protocols, reduced susceptibility to amphotericin B (MIC > 1 µg/mL) was observed in *A. flavus* (8.3%), *A. terreus* (22.7%), and section *Nidulantes* (32.1%). Acquired voriconazole resistance was found in 5.8% (9/156) of *A. fumigatus* isolates, including eight with *cyp51A* mutations (3 TR_34_/L98H, 4 TR_34_/L98H/S297T/F495I, and 1 TR_46_/Y121F/T289A). Voriconazole non-wild-type phenotypes were identified in 2.1% (3/158) of *A. flavus* isolates, including one carrying a novel P214L mutation (orthologous to *A. fumigatus* P216L). Members of the *Aspergillus niger* clade exhibited reduced susceptibility to itraconazole and isavuconazole. Anidulafungin, along with novel antifungals including orolofim, manogepix, and rezafungin, demonstrated broad activity across *Aspergillus* species, including those with intrinsic or acquired azole resistance. Ibrexafungerp also showed efficacy against azole-resistant *A. fumigatus* and *A. flavus*. In contrast, opelconazole exhibited limited activity against *A. flavus*, the *A. niger* clade, *Aspergillus sydowii*, and voriconazole-resistant *A. fumigatus*. These findings underscore the need for species-level identification, susceptibility testing of causative isolates, and continued surveillance to detect emerging resistance and support the use of novel non-azole antifungals for azole-resistant aspergillosis.

**IMPORTANCE:**

Timely and effective antifungal therapy is essential for aspergillosis. This multicenter surveillance study provides comprehensive insights into the species distribution and antifungal susceptibility of 550 clinical *Aspergillus* isolates in Taiwan, with intrinsic reduced susceptibility to amphotericin B, itraconazole, or isavuconazole noted in certain species. Regarding acquired resistance, a novel cyp51A mutation, P214L, was identified in an azole-resistant *Aspergillus flavus*, orthologous to *Aspergillus fumigatus* P216L. Recovery of azole-resistant *A. fumigatus* harboring TR34/L98H or TR46/Y121F/T289A mutations remains a concern and emphasizes the need for antifungal stewardship in the environment. Novel antifungals, including orolofim, manogepix, rezafungin, and ibrexafungerp, demonstrated broad activity across *Aspergillus* species, including resistant isolates. Nevertheless, the inhaled agent opelconazole exhibited limited activity against *A. flavus* regardless of voriconazole susceptibility and against other species showing reduced susceptibility to itraconazole. These findings highlight the importance of species-level identification, susceptibility testing, and continued surveillance and support the use of novel antifungals for aspergillosis.

## INTRODUCTION

Members of the genus *Aspergillus* are ubiquitous opportunistic fungal pathogens that produce dispersible conidia. They are implicated in diverse human diseases, including chronic pulmonary aspergillosis in patients with structural lung diseases and invasive aspergillosis, particularly in patients with immunocompromised conditions or pre-existing viral respiratory infections (1).

*Aspergillus* species associated with human infections include predominantly *Aspergillus fumigatus*, *Aspergillus flavus*, *Aspergillus terreus*, and the *Aspergillus niger* species complex (section *Nigri*) (1). These species exhibit inherited, species-specific susceptibility profiles to amphotericin B (AmB) or azole drugs, the two main classes used to treat aspergillosis (2). In addition, acquired azole resistance has emerged, posing a significant threat to treatment efficacy, particularly in *A. fumigatus*, where prolonged azole exposure, either through clinical treatment or environmental use, has driven the development of resistance (3). Given the limited availability of alternative antifungal agents, several novel drugs, including opelconazole, olorofim, rezafungin, manogepix, and ibrexafungerp, have been developed and are currently undergoing clinical evaluation (4).

Understanding the local epidemiology is essential for guiding effective therapy and designing molecular diagnostics to detect aspergillosis and associated resistance mutations. To address this need, the Taiwan Surveillance of Antimicrobial Resistance of Molds (TSARM), a biennial multicenter program established in 2016 by the Taiwan Mycology Reference Center (TMRC) at the National Health Research Institutes, investigates the molecular epidemiology and antifungal resistance trends of human-pathogenic filamentous fungi. In our earlier TSARM report, which characterized 492 clinical *Aspergillus* isolates collected during 2016–2020, we identified acquired azole resistance in *A. fumigatus* (5.9%), with all resistant isolates carrying TR_34_/L98H or TR_34_/L98H/S297T/F495I mutations in *cyp51A*, which are known azole-fungicide-related resistance mechanisms (3, 5). Taiwan is an island country with substantial azole fungicide use and frequent international horticultural trade, factors that may increase the potential to select for or introduce azole-resistant *A. fumigatus* of different lineages or with additional environmental resistance mutations. This earlier surveillance also detected acquired azole resistance in *A. flavus* (1.9%), including one isolate harboring a novel G441S mutation, orthologous to *A. fumigatus* G448S, a mutation associated with long-term azole therapy (5, 6). Given the ongoing selective pressure from both medical and agricultural azoles, periodic surveillance is essential for monitoring changes in resistance rates and emerging resistance mechanisms. Assessing the activity of novel antifungal agents is also critical, as treatment options for resistant isolates remain limited, particularly in cases of pneumonia or sinusitis caused by *A. flavus*, which shows reduced susceptibility to AmB along with acquired azole resistance. To meet these needs, the present study examined 550 subsequent clinical *Aspergillus* isolates collected during 2021–2023 through TSARM and surveillance programs supported by the Taiwan Centers for Disease Control (CDC). This work updates the epidemiology of clinical *Aspergillus* in Taiwan and assesses the *in vitro* activity of novel antifungals, providing essential data to inform clinical antifungal therapy.

## MATERIALS AND METHODS

### Isolate collection

Twenty-one hospitals, including 11 medical centers and 10 regional hospitals in Taiwan, participated in the TSARM and/or CDC programs. Briefly, *Aspergillus* isolates were obtained from diverse clinical specimens submitted for routine diagnostic fungal culture. In the TSARM program, isolates were collected from 20 hospitals between July and September 2022. In the CDC program, non-duplicated consecutive isolates (up to five per hospital per month) were collected from 10 hospitals between March and November of 2021 and 2023. All isolates were sent to the TMRC for further analysis.

### Species identification

*Aspergillus* species were identified based on morphological characteristics and calmodulin sequence analysis. For molecular identification, *Aspergillus* isolates were cultured on Sabouraud dextrose agar (SDA) for 3–7 days at 35°C. DNA was then extracted from the mycelia using the Quick-DNA Fungal/Bacterial Miniprep Kit (Zymo Research Corp.). Calmodulin sequencing was performed using polymerase chain reaction (PCR) with the primer pairs CF1 (forward, 5′-GCC GAC TCT TTG ACY GAR GAR-3′) and CF4 (reverse, 5′-TTT YTG CAT CAT RAG YTG GAC-3′) (7). PCR conditions were as follows: initial denaturation at 95°C for 5 min, followed by 35 cycles of 98°C for 15 s, 55°C for 30 s, and 72°C for 50 s, with a final extension at 72°C for 5 min, as previously described (8). For species identification, the amplified calmodulin sequences were compared with those in the National Center for Biotechnology Information Nucleotide Collection Database using the Basic Local Alignment Search Tool (BLAST). When BLAST results matched multiple species, phylogenetic analysis was performed to determine the final species assignment. A phylogenetic tree was constructed using concatenated calmodulin sequences from the study isolates and reference strains included in previous taxonomic studies (7, 9), and the maximum likelihood method was applied using MEGA X (http://www.megasoftware.net/). Each study isolate was assigned to a species based on either (i) calmodulin sequence identity to a single recognized species by BLAST or (ii) phylogenetic clustering with reference strains of a recognized species.

### Antifungal susceptibility testing

All isolates collected in 2021 and 2022 were tested against six conventional antifungal drugs: AmB, itraconazole, voriconazole, posaconazole, isavuconazole (all from Sigma-Aldrich, Missouri, USA), and anidulafungin (Toronto Research Chemicals, Canada). For isolates collected in 2023, *A. fumigatus*, *A. flavus*, and *A. terreus* were initially screened for azole resistance using SDA plates supplemented with itraconazole (2 μg/mL), voriconazole (1 μg/mL), posaconazole (0.25 μg/mL), and isavuconazole (2 μg/mL), respectively, as previously described (10). Isolates exhibiting growth on any azole-containing agar plate, isolates of rare species, and randomly selected isolates of other species (proportional to species distribution) were tested against six conventional and four novel antifungal agents: olorofim (ProbeChem, China), opelconazole, rezafungin, and manogepix (MedChemExpress, USA). Voriconazole-resistant *A. fumigatus* and *A. flavus*, as well as rare species collected in 2021–2022, were also tested against the four novel agents. All voriconazole-resistant *A. fumigatus* and *A. flavus* isolates were additionally tested against ibrexafungerp (MedChemExpress, USA).

Antifungal susceptibility testing was conducted following the CLSI M38-A3 broth microdilution method (11). In brief, stock solutions were prepared in dimethyl sulfoxide and diluted in RPMI medium using twofold serial dilutions. Conidial suspensions from 3- to 5-day-old potato dextrose agar cultures were adjusted to 0.2–0.5 McFarland at 530 nm and diluted in RPMI to obtain a twofold concentration of the final required inoculum. Final test concentrations were as follows: AmB, 0.015–8 μg/mL; azoles, 0.03–16 μg/mL; anidulafungin, 0.004–8 μg/mL; olorofim, manogepix, and rezafungin, 0.004–4 μg/mL; and ibrexafungerp, 0.015–16 μg/mL. The final inoculum density (0.4 × 10^4^ to 5 × 10^4^ CFU/mL) was verified by quantitative colony counts. The susceptibilities of AmB, azoles, and olorofim were interpreted based on minimum inhibitory concentrations (MICs), defined as the lowest concentration that prevents any discernible growth (100% inhibition) after 48 h at 35°C, whereas the susceptibilities of anidulafungin, rezafungin, manogepix, and ibrexafungerp were interpreted based on minimum effective concentrations (MECs), defined as the lowest concentration inducing compact hyphal growth after 24 h (4, 11). Quality control strains included *A. fumigatus* ATCC MYA-3626 and *Candida parapsilosis* ATCC 22,019.

Non-wild-type (non-WT) isolates of *A. fumigatus*, *A. flavus*, *A. terreus*, and *A. niger* species complex were classified based on the epidemiological cutoff values (ECVs) specified in the CLSI M57S (12). *A. fumigatus* was classified as voriconazole-resistant using the clinical breakpoint for resistance (≥2 µg/mL) defined in CLSI M38M51S (13). For convenience, voriconazole non-wild-type *A. flavus* isolates are also referred to as voriconazole-resistant in this study.

### *cyp51A* analysis and microsatellite genotyping

For voriconazole-resistant *A. fumigatus* and *A. flavus* isolates, the *cyp51A* gene was analyzed as previously described (14, 15), using *A. fumigatus* CM-237 (AF338659.1) and *A. flavus* NRRL3357 (XM_041289631.1) as reference sequences. Microsatellite genotyping of *A. fumigatus* was performed using nine short tandem repeat (STR) markers (16). A phylogenetic dendrogram was constructed using the unweighted pair group method with arithmetic mean in BioNumerics version 8.1 (Applied Maths, Belgium), incorporating clinical and environmental azole-resistant *A. fumigatus* isolates harboring environmental resistance mutations from our previous reports (5, 10, 17), as well as isolates from various countries (Table S1).

## RESULTS

### Species distributions

**Fig 1:**
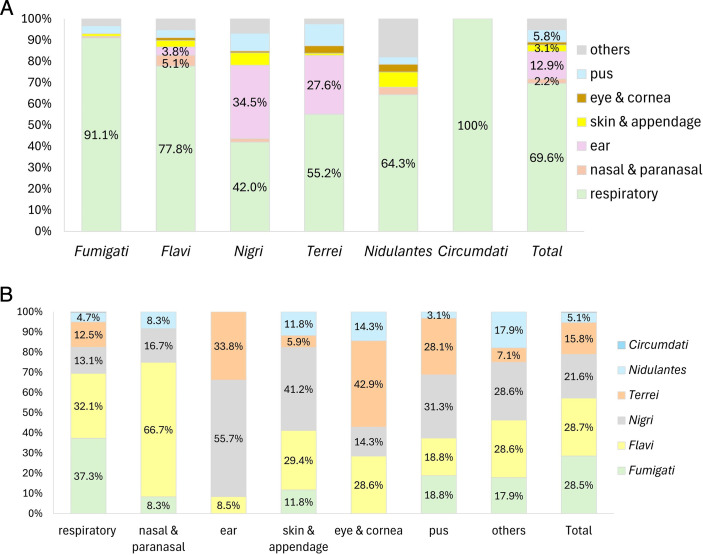
(**A**) Specimen types, stratified by *Aspergillus* section. (**B**) Distribution of *Aspergillus* sections across different clinical specimen types among 550 clinical *Aspergillus* isolates.

A total of 550 *Aspergillus* isolates were collected, which comprised 119, 231, and 200 isolates from 2021, 2022, and 2023, respectively. The majority were obtained from the respiratory tract (69.6%), followed by the ear (12.9%), unspecified pus specimens (5.8%), skin and appendages (3.1%), the nasal cavity or paranasal sinuses (2.2%), the eye or cornea (1.3%), and others (5.1%) (Table 1; Fig. 1A). Species identification of 531 isolates was supported by calmodulin sequence alignment or phylogenetic analysis with >99.5% identity to reference species. The remaining 13 isolates were identified through phylogenetic analysis, demonstrating sequence identities of 98.3%–99.4% to their respective reference species within the same phylogenetic cluster (Table S2). A total of 24 species, belonging to six *Aspergillus* sections, were identified: *Fumigati* (28.5%), *Flavi* (28.7%), *Nigri* (21.6%), *Terrei* (15.8%), *Nidulantes* (5.1%), and *Circumdati* (0.2%).

**TABLE 1:** Species distribution and isolation sites of 550 clinical *Aspergillus* isolates^*a*^

Section/Species	Isolate no. (%)	Specimen type (no., %)
Section *Fumigati*	157 (28.5%)	Respiratory (143, 91.1%), nasal (1, 0.6%), and ear (0, 0%)
*A. fumigatus*	156 (28.4%)	Sputum (128), BALF (6), ETA (6), pus (6), unspecified tissue (5), lung tissue (2), nail (1), nasal sinus (1), and leg (1)
*A. hiratsukae*	1 (0.2%)	BALF (1)
Section *Flavi*	158 (28.7%)	Respiratory (123, 77.8%), nasal (8, 5.1%), and ear (5, 3.2%)
*A. flavus*	145 (26.4%)	Sputum (102), nasal sinus (8), BALF (6), ear (6), pus (4), ETA (4), unspecified tissue (4), nail (3), cornea (2), lung tissue (2), skin (2), blood (1), and unknown (1)
*A. tamarii*	12 (2.2%)	Sputum (8), urine (2), CAPD wound (1), and pus (1)
*A. pseudonomiae*	1 (0.2%)	Sputum (1)
Section *Nigri*	119 (21.6%)	Respiratory (50, 42.0%), nasal (2, 1.7%), and ear (41, 34.5%)
*A. welwitschiae*	68 (12.4%)	Ear (33), sputum (17), pus (5), ETA (3), urine (3), BALF (2), nail (2), skin (1), nasal pus (1), and unknown (1),
*A. niger*	18 (3.3%)	Sputum (7), pus (3), ear (2), blood (1), ETA (1), lung tissue (1), nasal sinus (1), and urine (1)
*A. brunneoviolaceus*	12 (2.2%)	Sputum (8), BALF (2), nail (1), and skin (1)
*A. neoniger*	8 (1.5%)	Sputum (5) and ear (3)
*A. tubingensis*	8 (1.5%)	Pus (2), BALF (1), blood (1), conjunctiva (1), ear (1), lung tissue (1), and unspecified tissue (1)
*A. aculeatinus*	2 (0.4%)	Nail (1) and sputum (1)
*A. costaricaensis*	1 (0.2%)	Nail (1)
*A. japonicus*	1 (0.2%)	BALF (1)
*A. luchuensis*	1 (0.2%)	Ear (1)
Section *Terrei*	87 (15.8%)	Respiratory (48, 55.2%), nasal (0, 0%), and ear (24, 27.6%)
*A. terreus*	87 (15.8%)	Sputum (39), ear (24), pus (9), BALF (4), ETA (3), pleural fluid (2), cornea (2), unspecified tissue (2), nail (1), and eye (1)
Section *Nidulantes*	28 (5.1%)	Respiratory (18, 64.3%), nasal (1, 3.6%), and ear (0, 0%)
*A. nidulans*	5 (0.9%)	Sputum (5)
*A. spinulosporus*	3 (0.5%)	Sputum (3)
*A. unguis*	3 (0.5%)	Sputum (2) and urine (1)
*A. stellatus*	1 (0.2%)	BALF (1)
Series *Versicolores*		
*A. sydowii*	13 (2.4%)	Sputum (4), unspecified tissue (2), cornea (1), nail (1), nasal sinus (1), pleural fluid (1), pus (1), skin (1), and unknown (1)
*A. amoenus*	1 (0.2%)	CSF (1)
*A. austroafricanus*	1 (0.2%)	Sputum (1)
*A. versicolor*	1 (0.2%)	Sputum (1)
Section *Circumdati*	1 (0.2%)	Respiratory (1, 100%)
*A. subramanianii*	1 (0.2%)	Sputum (1)

^*a*^ BALF, bronchoalveolar lavage; CSF, cerebrospinal fluid; CAPD, continuous ambulatory peritoneal dialysis; ETA, endotracheal aspirate.

Section *Fumigati* was composed predominantly of *A. fumigatus* (99.4%) and one *Aspergillus hiratsukae* isolate. Section *Flavi* consisted mainly of *A. flavus* (91.8%), along with *Aspergillus tamarii* (7.7%) and one *Aspergillus pseudonomiae* isolate. Section *Terrei* was composed exclusively of *A. terreus* isolates. Section *Nigri* was composed predominantly of *Aspergillus welwitschiae* (syn. *Aspergillus awamori*; 57.1%), followed by *A. niger* (14.3%), *Aspergillus brunneoviolaceus* (10.1%), *Aspergillus neoniger* (6.7%), *Aspergillus tubingensis* (6.7%), and others. Section *Nidulantes* included primarily *Aspergillus sydowii* (46.4%) and *Aspergillus nidulans* (17.9%).

Sections *Fumigati* and *Flavi* comprised the majority of isolates recovered from respiratory specimens (37.3% and 32.1%, respectively). *A. flavus* accounted for two-thirds (66.7%) of isolates from nasal cavity or paranasal sinus specimens. In contrast, isolates from ear specimens were predominantly from sections *Nigri* and *Terrei* (57.7% and 33.8%, respectively; Fig. 1B).

### Antifungal susceptibility

All 350 isolates collected in 2021–2022 were tested against conventional antifungal drugs; 125 isolates, consisting of 14 collected in 2021–2022 and 111 collected in 2023, were tested against both conventional and novel antifungal agents.

**Fig 2:**
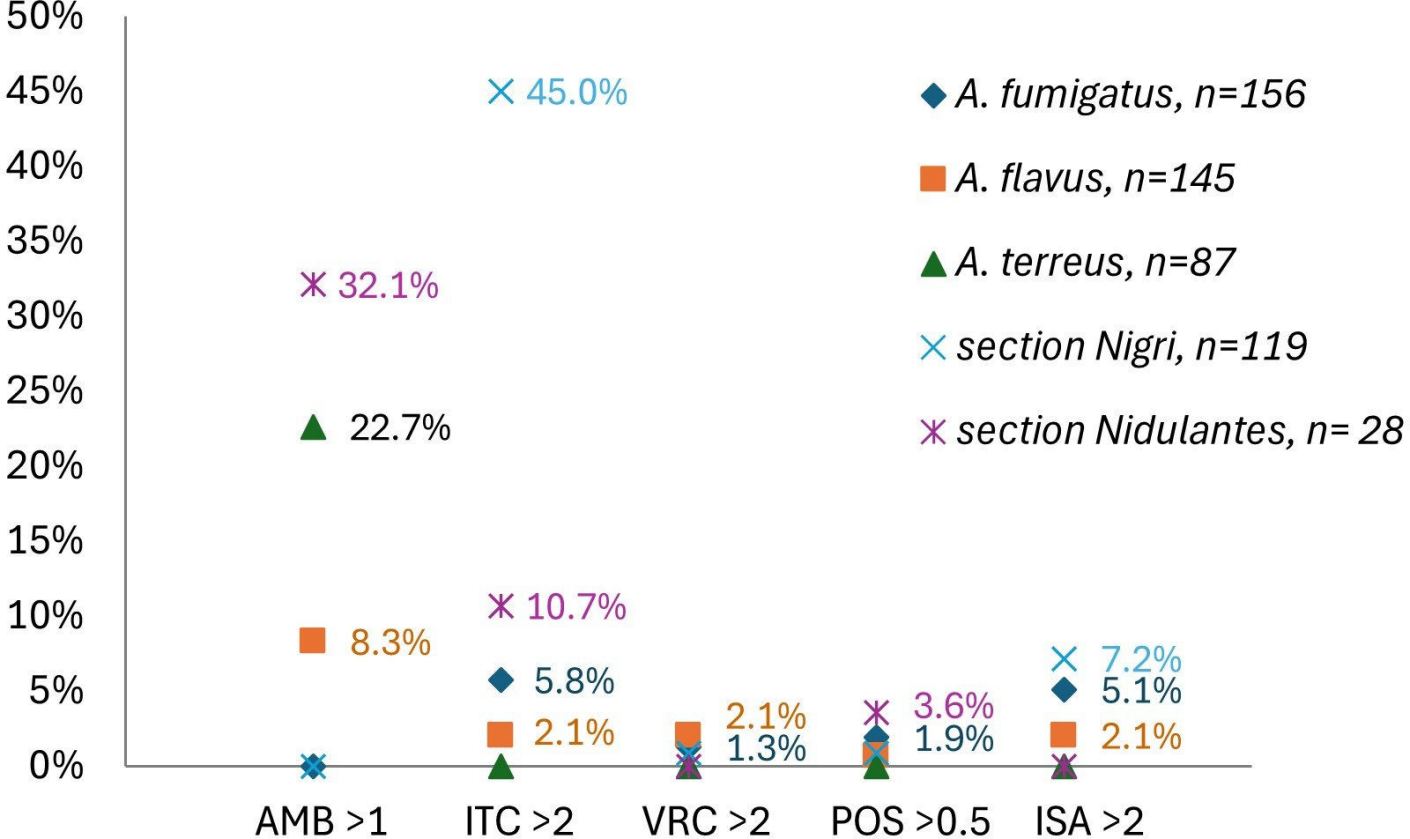
Proportion of *Aspergillus* species with specified MIC (µg/mL) categories against conventional antifungals.

All *A. fumigatus* and section *Nigri* isolates tested had AmB MICs ≤ 1 µg/mL, whereas 32.1%, 22.7%, and 8.3% of section *Nidulantes*, *A. terreus*, and *A. flavus* isolates, respectively, had MICs > 1 µg/mL (Table 2; Fig. 2). Most isolates demonstrated MICs ≤ 2 µg/mL for itraconazole, voriconazole, and isavuconazole and ≤0.5 µg/mL for posaconazole, with the following exceptions. Nine *A. fumigatus* isolates (5.8%, 9/156) were resistant to voriconazole and concurrently exhibited non-WT phenotypes for itraconazole and isavuconazole, along with reduced susceptibility to posaconazole. Three *A. flavus* isolates (2.1%, 3/145) demonstrated a voriconazole non-WT phenotype, accompanied by non-WT phenotypes for itraconazole, posaconazole, and isavuconazole (Table 3). Only 55% of section *Nigri* isolates exhibited itraconazole MICs ≤ 2 µg/mL, and all isolates with MICs >2 µg/mL belonged to *A. welwitschiae*, *A. niger*, *A. neoniger*, *A. tubingensis*, and *Aspergillus costaricaensis*, all of which have been classified as members of the *A. niger* clade within section *Nigri* in previous taxonomic studies (7). Among section *Nigri* isolates with itraconazole MICs ≥ 16 µg/mL, 24 (64.9%, 24/34) showed trailing growth with visible hyphae growth, typically initiating at concentrations of 2 or 4 µg/mL and persisting through 16 µg/mL. These species exhibited elevated geometric mean (GM) MICs for both itraconazole (2.49 µg/mL for *A. welwitschiae* and 6.06 µg/mL for *A. niger*) and isavuconazole (1.35 and 1.82 µg/mL, respectively). In contrast, *A. brunneoviolaceus*, *Aspergillus aculeatinus*, and *Aspergillus japonicus* (members of the *Aspergillus aculeatus* clade) (7) showed substantially lower AmB and azole MICs (Fig. S1). Additionally, *Aspergillus unguis* (section *Nidulantes*) and *Aspergillus subramanianii* (section *Circumdati*) exhibited reduced susceptibility to both AmB and itraconazole (Table 4). Anidulafungin demonstrated consistent activity across all isolates tested, with MECs ≤ 0.015 µg/mL.

**TABLE 2:** MICs and GM MICs of conventional antifungal agents against *Aspergillus* isolates from 2021 to 2023^*b*^

Antifungal agents		MIC (μg/mL)											non-WT no. (%)
Section/species (no. of isolates tested)		≤0.03	0.06	0.12	0.25	0.50	1	2	4	8	≥16	GM	
AmB													
*Fumigati*	*A. fumigatus* (124/**156**)	1		1	8	68	46					0.60	0
*Flavi*	*A. flavus* (108/**145**)					20	79	8	1			0.94	0
	*A. tamarii* (12)				5	5	2					0.42	
*Terrei*	*A. terreus* (75/**87**)					14	44	15	2			1.05	0
*Nigri*	*A. welwitschiae* (63)		1	4	37	19	2					0.30	
	*A. niger* (15)			6	6	3						0.21	0
	*A. brunneoviolaceus* (12)	5	4	3								0.05	
*Nidulantes*	*A. sydowii* (13)					3	3	7				1.24	
Itraconazole													
*Fumigati*	*A. fumigatus* (124/**156**)		1	22	58	14	18	2			9	0.42	11 (**7.1**)
*Flavi*	*A. flavus* (108/**145**)		6	3	40	26	26	4	1		2	0.45	7 (**4.8**)
	*A. tamarii* (12)		2	6	4							0.14	
*Terrei*	*A. terreus* (75/**87**)	12	22	24	11	4	2					0.10	0
*Nigri*	*A. welwitschiae* (63)				9	6	16	6	9		17 (**13**)^*a*^	2.49	
	*A. niger* (15)				1	1		5	1		7 (**1**)^*a*^	6.06	7 **(46.7)**
	*A. brunneoviolaceus* (12)	10	2									0.03	
*Nidulantes*	*A. sydowii* (13)				4	2	2	5				0.77	
Voriconazole													
*Fumigati*	*A. fumigatus* (124/**156**)			2	10	87	16	7	1		1	0.57	9 (**5.8**)
*Flavi*	*A. flavus* (108/**145**)					3	46	56	3			1.46	3 (**2.1**)
	*A. tamarii* (12)						10	2				1.12	
*Terrei*	*A. terreus* (75/**87**)			2	7	36	26	4				0.62	0
*Nigri*	*A. welwitschiae* (63)				1	18	42	2				0.82	
	*A. niger* (15)					1	9	5				1.20	0
	*A. brunneoviolaceus* (12)	1	5	5	1							0.09	
*Nidulantes*	*A. sydowii* (13)					3	5	5				1.11	
Posaconazole													
*Fumigati*	*A. fumigatus* (124/**156**)	4	63	42	7	5	3					0.09	
*Flavi*	*A. flavus* (108/**145**)		8	31	61	7					1	0.20	1 (**0.7**)
	*A. tamarii* (12)		8	3	1							0.08	
*Terrei*	*A. terreus* (75/**87**)	9	42	23	1							0.07	0
*Nigri*	*A. welwitschiae* (63)	1	5	28	26	3						0.16	
	*A. niger* (15)			3	10	2						0.24	0
	*A. brunneoviolaceus* (12)	10	2									0.03	
*Nidulantes*	*A. sydowii* (13)			1	9	3						0.28	
Isavuconazole													
*Fumigati*	*A. fumigatus* (124/**156**)				1	66	46	3	3		5	0.83	1 (**7.1**)
*Flavi*	*A. flavus* (108/**145**)				1	5	87	12	2	1		1.08	15 (**10.3**)
	*A. tamarii* (12)				1	8	3					0.56	
*Terrei*	*A. terreus* (75/**87**)		1	1	9	32	32					0.59	0
*Nigri*	*A. welwitschiae* (63)					3	32	26	2			1.35	
	*A. niger* (15)					1		14				1.82	0
	*A. brunneoviolaceus* (12)	1	5	5	1							0.09	
*Nidulantes*	*A. sydowii* (13)				2	4	6	1				0.69	

^*a*^ The number in parentheses refers to the number of isolates exhibiting trailing growth.

^*b*^ Dark-shaded cells indicate voriconazole resistance in *A. fumigatus* and non-WT phenotypes in others. Bold numbers in parentheses show total isolates, including those screened for azole resistance.

**TABLE 3:** Laboratory characteristics of voriconazole-resistant *A. fumigatus* and non-WT *A. flavus* isolates from 2021 to 2023^*b*^

Strain	Year	Source	Cyp51A substitutions	MIC (μg/mL)							MEC (μg/mL)			
				AmB	ITC	VRC	POS	ISA	OPC	OLF	AND	IBX	MGX	RZF
*A. fumigatus*														
01-008	2021	Sputum	TR_34_/L98H	0.25	>16	2	0.5	4	>16	0.06	≤0.004	0.06	0.03	0.015
03-009	2021	Sputum	TR_34_/L98H	0.5	>16	2	0.5	4	>16	0.06	≤0.004	0.06	0.03	0.015
C04-006	2022	Sputum	TR_34_/L98H/S297T/F495I	1	>16	4	0.5	>16	>16	0.06	≤0.004	0.06	0.03	0.06
C03-014	2022	Sputum	TR_46_/Y121F, T289A	1	>16	>16	0.5	>16	>16	0.06	≤0.004	0.06	0.03	0.03
S07-020	2022	Pus	polymorphism^*a*^	0.5	>16	2	0.25	2	>16	0.12	≤0.004	0.06	0.06	0.015
S02-057	2022	Sputum	TR_34_/L98H/S297T/F495I	0.5	>16	2	1	>16	>16	0.12	≤0.004	0.06	0.06	0.015
S06-004	2023	Sputum	TR_34_/L98H/S297T/F495I	0.5	>16	2	1	>16	>16	0.06	≤0.004	0.06	0.015	0.015
S07-031	2023	Sputum	TR_34_/L98H/S297T/F495I	0.25	>16	2	1	>16	>16	0.06	≤0.004	0.06	0.03	0.015
N02-005	2023	Sputum	TR_34_/L98H	0.5	>16	2	0.5	4	>16	0.06	≤0.004	0.06	0.06	0.015
*A. flavus*														
S06-009	2021	Sputum	T329A	1	>16	4	0.5	8	>16	0.12	≤0.004	0.25	0.03	0.03
N03-008	2023	Sputum	A199T	1	4	4	0.5	4	>16	0.03	≤0.004	0.06	0.008	0.03
S06-021	2023	Ear pus	P214L	1	>16	4	>16	4	>16	0.12	0.015	0.015	0.06	≤0.004

^*a*^ F46Y/M172V/N248T/D255E/E427K; no mutation in the promoter region.

^*b*^ AND, anidulafungin; IBX, ibrexafungerp; ISA, isavuconazole; ITC, itraconazole; MGX, manogepix; OLF, olorofim; OPC, opelconazole; POS, posaconazole; RZF, rezafungin; VRC, voriconazole.

**TABLE 4:** MICs/MECs of antifungal agents against uncommon *Aspergillus* species^*c*^

		MIC or MEC (GM) (μg/mL)^*b*^									
Section	Species (no. of isolates tested^*a*^)	Conventional antifungals						Novel antifungals			
		AmB	ITC	VRC	POS	ISA	AND	OPC	OLF	MGX	RZF
*Fumigati*	*A. hiratsukae* (1, 1)	1	0.25	1	0.25	1	≤0.004	0.5	≤0.004	0.008	0.008
*Flavi*	*A. pseudonomiae* (1,1)	1	0.5	2	0.25	1	0.008	>16	0.03	0.015	0.06
*Nigri*	*A. neoniger* (8, 1)	0.12−1 (0.27)	4 to >16 (20.75)	1−2 (1.41)	0.12−0.5 (0.21)	2−4 (2.18)	≤0.004	>16	0.06	0.08	0.008
	*A. tubingensis* (8, 2)	0.12−0.5 (0.25)	1 to >16 (19.03)	1 to >16 (2)	0.25−1 (0.35)	2 to >16 (3.67)	≤0.004	>16	0.03, 0.06	≤0.004, 0.008	0.008
	*A. aculeatinus* (2, 1)	0.06	≤0.03, 0.06	0.06	≤0.03, 0.06	0.25	≤0.004	≤0.03	0.015	≤0.004	0.008
	*A. costaricaensis* (1,1)	0.25	>16	2	0.25	4	≤0.004	>16	0.06	≤0.004	0.015
	*A. japonicus* (1,1)	0.03	0.06	0.12	0.06	0.25	≤0.004	≤0.03	0.008	0.008	≤0.004
	*A. luchuensis* (1,1)	0.25	0.5	0.5	0.12	1	≤0.004	>16	0.03	≤0.004	≤0.004
*Nidulantes*	*A. nidulans* (5, 2)	0.5−2 (1)	0.06−0.25 (0.11)	0.06−0.25 (0.14)	0.06−0.12 (0.08)	0.06−0.25 (0.14)	≤0.004	≤0.03	0.06	0.06, 0.008	0.015, 0.008
	*A. spinulosporus* (3, 2)	0.5−1	0.25−1	0.5	0.25	0.12−0.25	≤0.004−0.008	>16	0.015, 0.03	0.015, 0.03	0.03, 0.015
	*A. unguis* (3, 2)	1−2	4 to >16	0.5	0.5−1	1−2	≤0.004−0.008	>16	0.008	0.06, 0.03	≤0.004, 0.015
	*A. amoenus* (1,1)	1	0.25	0.5	0.12	0.5	≤0.004	>16	≤0.004	≤0.004	0.008
	*A. austroafricanus* (1,1)	0.5	0.12	0.25	0.12	0.12	≤0.004	≤0.03	≤0.004	≤0.004	≤0.004
	*A. versicolor* (1,1)	1	0.06	0.12	0.06	0.12	≤0.004	≤0.03	0.008	0.008	≤0.004
	*A. stellatus* (1,1)	1	2	0.5	0.5	0.25	0.008	>16	0.008	0.03	0.06
*Circumdati*	*A. subramanianii* (1,1)	2	8	2	0.5	2	≤0.004	>16	0.03	0.015	0.015

^*a*^ The first and second numbers in the parentheses indicate the no. of isolates tested for conventional and novel antifungals, respectively.

^*b*^ For isolate numbers greater than three, the GM MICs are provided in parentheses. If all isolates in the cell share the same MIC, a single MIC value is displayed.

^*c*^ AND, anidulafungin; IBX, ibrexafungerp; ISA, isavuconazole; ITC, itraconazole; MGX, manogepix; OLF, olorofim; OPC, opelconazole; POS, posaconazole; RZF, rezafungin; VRC, voriconazole.

For the novel antifungal agents (Tables 4 and 5), olorofim exhibited MICs ≤ 0.12 µg/mL against all tested isolates. Manogepix and rezafungin demonstrated MECs ≤ 0.06 µg/mL across all isolates, except for one *A. flavus* isolate with a manogepix MEC of 0.12 µg/mL. Low ibrexafungerp MECs (≤0.25 µg/ml) were observed for voriconazole-resistant *A. fumigatus* and *A. flavus* isolates. In contrast, opelconazole displayed species- and strain-specific variability. High opelconazole MICs (≥8 µg/mL, most >16 µg/mL) were observed in 13 *A. fumigatus* isolates (including all nine voriconazole-resistant isolates), in most *A. flavus* isolates (92.6%, 25/27) regardless of voriconazole susceptibility, in *A. pseudonomiae* (section *Flavi*), in all isolates of the *A. niger* clade (including *Aspergillus luchuensis*), and in certain species within section *Nidulantes* (*A. sydowii*, *Aspergillus spinulosporus*, *A. unguis*, *Aspergillus amoenus*, and *Aspergillus stellatus*), as well as in *A. subramanianii*. In contrast, low opelconazole MICs (≤ 2 µg/mL) were observed in 83.3% (20/24) of voriconazole-susceptible *A. fumigatus* isolates, 96% of *A. terreus* isolates, the *A. aculeatus* clade within section *Nigri*, and certain species within section *Nidulantes.*

**TABLE 5:** MICs/MECs and GM MICs/MECs of novel antifungal agents against *Aspergillus* isolates from 2023, and anidulafungin against *Aspergillus* isolates from 2021 to 2023^*c*^

Antifungal agents		MIC or MEC (μg/mL)										
Section/species (no. of isolates tested)												
Opelconazole		≤0.03	0.06	0.12	0.25	0.50	1	2	4	8	≥16	GM
*Fumigati*	*A. fumigatus* (33)	8	3	1	1	3	2	2		1	12^*a*^	1.094
*Flavi*	*A. flavus* (27)		2							1	24^*a*^	19.092
	*A. tamarii* (1)		1									0.060
*Terrei*	*A. terreus* (25)	18	4			1		1		1		0.055
*Nigri*	*A. welwitschiae* (10)									1	9	27.858
	*A. niger* (6)			1							5	12.613
	*A. brunneoviolaceus* (2)	2										**≤** 0.030
*Nidulantes*	*A. sydowii* (3)^*b*^										3	32.000
Olorofim		≤0.004	0.008	0.015	0.03	0.06	0.12					GM
*Fumigati*	*A. fumigatus* (33)			5	15	11	2					0.037
*Flavi*	*A. flavus* (27)			6	10	8	3					0.037
	*A. tamarii* (1)				1							0.030
*Terrei*	*A. terreus* (25)	11	11	3								0.006
*Nigri*	*A. welwitschiae* (10)				3	7						0.049
	*A. niger* (6)				1	5						0.053
	*A. brunneoviolaceus* (2)	1		1								0.008
*Nidulantes*	*A. sydowii* (1)	1										**≤** 0.004
Manogepix		≤0.004	0.008	0.015	0.03	0.06	0.12					GM
*Fumigati*	*A. fumigatus* (33)		2	3	18	10						0.032
*Flavi*	*A. flavus* (27)		6	6	9	5	1					0.023
	*A. tamarii* (1)		1									0.008
*Terrei*	*A. terreus* (25)		7	12	3	3						0.016
*Nigri*	*A. welwitschiae* (10)		9		1							0.009
	*A. niger* (6)	4	2									0.005
	*A. brunneoviolaceus* (2)	2										**≤** 0.004
*Nidulantes*	*A. sydowii* (1)	1										**≤** 0.004
Rezafungin		≤0.004	0.008	0.015	0.03	0.06	0.12					GM
*Fumigati*	*A. fumigatus* (33)		2	17	12	2						0.020
*Flavi*	*A. flavus* (27)	2	4	6	9	6						0.021
	*A. tamarii* (1)				1							0.030
*Terrei*	*A. terreus* (25)	21	3	1								0.005
*Nigri*	*A. welwitschiae* (10)	7	3									0.005
	*A. niger* (6)	6										**≤** 0.004
	*A. brunneoviolaceus* (2)		2									0.008
*Nidulantes*	*A. sydowii* (1)		1									0.008
Anidulafungin		≤0.004	0.008	0.015	0.03	0.06	0.12					GM
*Fumigati*	*A. fumigatus* (124)	108	10	6								0.005
*Flavi*	*A. flavus* (108)	95	7	6								0.005
	*A. tamarii* (12)	12										**≤** 0.004
*Terrei*	*A. terreus* (75)	74	1									**≤** 0.004
*Nigri*	*A. welwitschiae* (63)	63										**≤** 0.004
	*A. niger* (15)	15										**≤** 0.004
	*A. brunneoviolaceus* (12)	10										**≤** 0.004
*Nidulantes*	*A. sydowii* (13)	13										**≤** 0.004

^*a*^ Includes nine voriconazole-resistant *A. fumigatus* isolates and three voriconazole non-WT *A. flavus* isolates.

^*b*^ Additional two isolates from 2021 to 2022 were tested.

^*c*^ Shaded cells indicate table/section headings. The values shown are MICs for opleconazole and olorofim, and MECs for manogepix, rezafungin, and anidulafungin.

### *cyp51A* analysis and microsatellite genotyping

**Fig 3:**
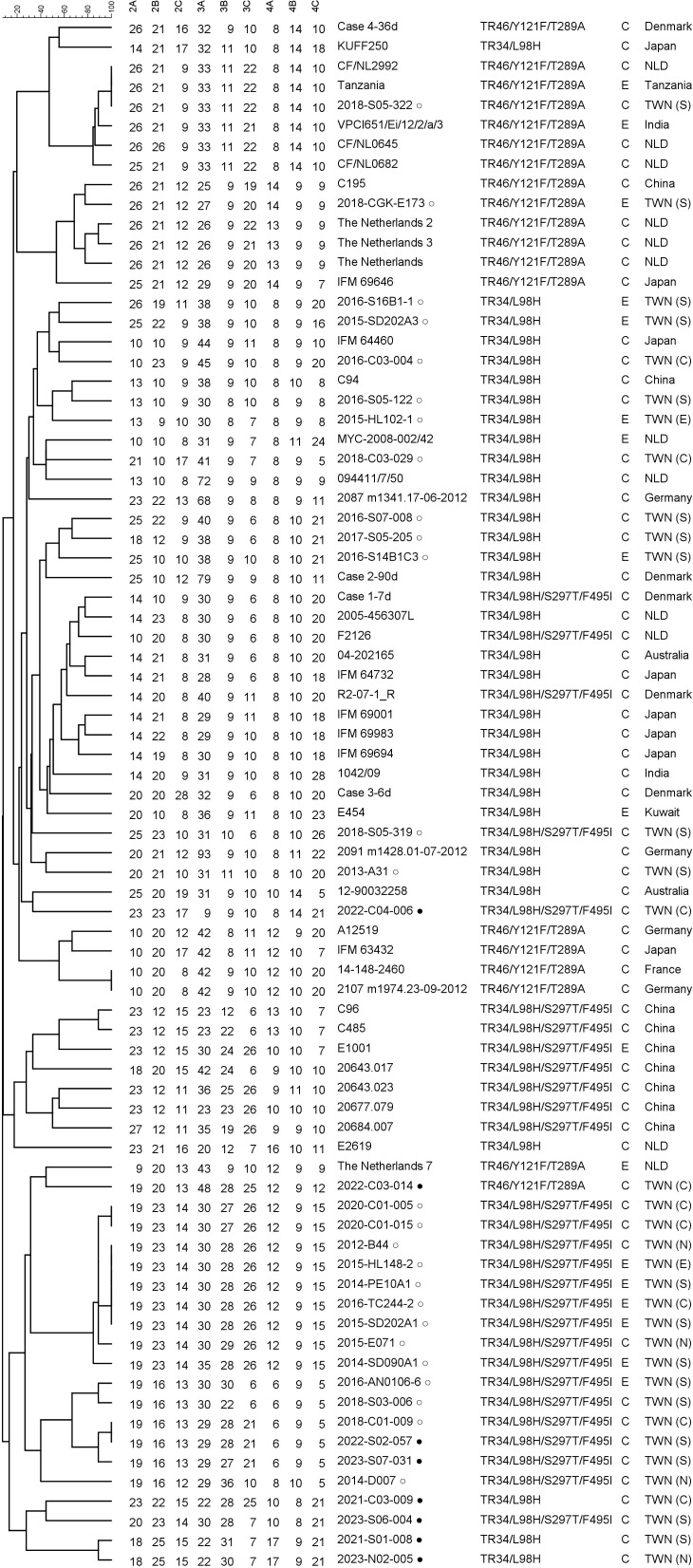
Phylogenetic dendrogram of azole-resistant *Aspergillus fumigatus* based on microsatellite genotyping. Cyp51A substitutions are indicated. Isolates include those from this study (●), previous Taiwanese isolates (○), and isolates from overseas countries. C, clinical; E, environmental; TWN (N)/(C)/(S)/(E), northern/central/southern/eastern Taiwan; NLD, the Netherlands.

Of nine voriconazole-resistant *A. fumigatus* isolates, 3, 4, and 1 isolate harbored the TR_34_/L98H, TR_34_/L98H/S297T/F495I, and TR_46_/Y121F/T289A mutations, respectively. Microsatellite genotyping revealed that two isolates (2022-S02-057 and 2023-S07-031) carrying TR_34_/L98H/S297T/F495I mutations shared nearly identical STR profiles with a preexisting microsatellite genotype from an isolate collected in 2018 (2018-C01-009) (5) (Fig. 3). The remaining six isolates, including the one carrying TR_46_/Y121F/T289A mutations, did not cluster with any strains from our earlier collection (5, 10, 17). Among three voriconazole-resistant *A. flavus* isolates, *cyp51A* substitutions T329A, A199T, and P214L were identified, respectively.

## DISCUSSION

This study provides updated multicenter surveillance data on the epidemiology of clinical *Aspergillus* isolates in Taiwan, which is located in subtropical to tropical climate regions. Sections *Fumigati*, *Flavi*, *Terrei*, and *Nigri* collectively accounted for most clinical isolates, consistent with global findings (1). Notably, section *Flavi* comprised a much higher proportion in Taiwan (28.7%) than in temperate countries, including France, Italy, Canada, Japan, and Australia (3%–17.3%) (18–22). Similarly elevated proportions have been reported in other subtropical or tropical countries such as Indonesia (73.4%), India (46.9%), Thailand (27.9%), and Mexico (25.3%), as well as in arid countries such as Iran (50.2%) (22–26) (Table S3; Fig. S2). The prevalence of *A. flavus* in these regions may reflect its greater ability to survive in hot or arid environments (27).

Isolation sites varied by *Aspergillus* species. *A. fumigatus* and *A. flavus* predominated in respiratory specimens, consistent with their well-established role in pulmonary aspergillosis (2, 27). Section *Nigri*, predominantly *A. welwitschiae*, was most frequently recovered from ear specimens, in line with the recognition of this section as the major causative agent of otomycosis globally (28). This may be attributable to its environmental abundance, tendency to intermix with dust and air particulates, and inherent enzymatic (e.g., phospholipase) and biofilm-forming capabilities, which facilitate penetration and persistence in the external ear canal (29, 30). Notably, *A. welwitschiae* was also the most common isolated species among *Aspergillus* otomycosis cases in China (36.2%, 25/69) and Iran (60.5%, 52/86) (31, 32). The higher recovery of *A. flavus* from nasal sinus specimens in our study aligns with Taiwan’s subtropical–tropical climate and the larger size of *A. flavus* spores (3−7 µm), which tend to be deposited in the nasal cavity and sinuses, whereas the smaller *A. fumigatus* spores (2.5−3.5 µm) more readily reach the lower respiratory tract (33).

AmB and azole agents (primarily voriconazole) are the main treatments for aspergillosis, with echinocandins serving as alternative options (2). This study demonstrated species-specific antifungal susceptibility patterns, including both intrinsic and acquired resistance in certain *Aspergillus* species. *A. flavus*, *A. terreus*, and sections *Nidulantes* and *Circumdati* exhibited reduced susceptibility to AmB, consistent with previous reports (2, 34). Conventional azoles were generally active *in vitro*; however, reduced susceptibility to itraconazole and isavuconazole was observed among the *A. niger* clade isolates. Of note, the interpretation of itraconazole MICs remains challenging. Paradoxical growth, defined as increased growth occurring at least two dilutions above the MIC, and itraconazole tolerance, characterized by hyphal extension or conidial germination above the MIC on microscopic examination, have been described (35, 36). In this study, trailing growth was also frequently observed among the *A. niger* clade isolates. Such growth phenomena may introduce variability in itraconazole MIC determination among laboratory personnel. Moreover, the Etest and Sensititre YeastOne assays have failed to detect high itraconazole MICs in section *Nigri* isolates that are evident using reference EUCAST or CLSI methods (37, 38). These factors contribute to variation in reported resistance trends. Nevertheless, even when trailing growth was disregarded, *A. niger* clade isolates still showed itraconazole and isavuconazole MICs about two dilutions higher than those of *A. fumigatus*, *A. flavus*, and *A. terreus* in this and previous TSARM studies (5). The ESCMID/ECMM/ERS guidelines recommend avoiding itraconazole and isavuconazole for infections caused by the *A. niger* species complex due to elevated MICs (2); however, supporting clinical evidence remains limited, and further studies are warranted. In contrast, the *A. aculeatus* clade (e.g., *A. brunneoviolaceus*) showed high azole susceptibility, highlighting the therapeutic value of azoles and the relevance for accurate phylogenetic classification within section *Nigri*.

The voriconazole resistance rate in *A. fumigatus* observed in this study was comparable to that reported in the 2016−2020 surveillance (5.8% vs. 4.2%, *P* = 0.783), and the rate of non-WT voriconazole phenotypes in *A. flavus* was similarly consistent (2.1% vs. 1.9%, *P* = 1.000), whereas *A. terreus* isolates with acquired azole resistance have not yet been identified (Table S4) (5). The azole resistance rates (<10%) among the three major *Aspergillus* species suggest that azole monotherapy may be considered for the empirical treatment of pulmonary aspergillosis in Taiwan (3); however, azole resistance should be suspected in cases of azole treatment failure. Notably, most azole-resistant *A. fumigatus* isolates harbored environmental mutations, namely TR_34_/L98H and TR_46_/Y121F/T289A, which are thought to be driven by azole fungicide use (3). Further microsatellite genotyping identified emerging novel genotypes among isolates with TR_34_/L98H, TR_34_/L98H/S297T/F495I, and TR_46_/Y121F/T289A mutations, along with clonal expansion of a preexisting TR_34_/L98H/S297T/F495I genotype in Taiwan (5). These findings indicate ongoing local evolution of azole-resistant *A. fumigatus*, underscoring the need for continued surveillance and environmental antifungal stewardship.

One azole-non-WT *A. flavus* isolate harbored a P214L mutation in *cyp51A*, corresponding to the P216L mutation in *A. fumigatus*. The P216L mutation has been reported in an *A. fumigatus* isolate recovered from an aspergilloma after 1 year of itraconazole therapy and was shown, via gene replacement, to confer resistance to both itraconazole and posaconazole (39, 40). Subsequently, a Spanish study identified an *A. flavus* isolate with the P214L mutation, also exhibiting resistance to both itraconazole and posaconazole (41). Here, we report an additional azole-resistant *A. flavus* isolate harboring the same mutation, further supporting its role in azole resistance. However, our isolate displays a pan-azole-resistant phenotype, suggesting that additional mechanisms may contribute to its voriconazole resistance. The role of a T329A substitution identified in another azole-non-WT *A. flavus* isolate (corresponding to position 335 in *A. fumigatus*) remains unclear, as this mutation has not been associated with azole resistance in either species. Additionally, A199T has been identified in both azole-susceptible and azole-resistant *A. flavus* isolates (5).

Opelconazole is a novel inhaled triazole antifungal designed for targeted pulmonary delivery (4). Consistent with previous data, opelconazole demonstrated *in vitro* activity against *A. fumigatus* and *A. terreus* isolates, except *A. fumigatus* harboring TR_34_/L98H or TR_46_/Y121F/T289A mutations (42). Limited data on non-*fumigatus Aspergillus* species showed elevated MICs (6 to >8 µg/mL) for three *A. flavus* strains (including ATCC 204,034) and *A. niger* ATCC 1015 (42). Our findings align with and expand upon these observations, highlighting species-specific variability in opelconazole susceptibility, with reduced susceptibility observed in *A. flavus* and in species exhibiting decreased susceptibility to itraconazole, for which the underlying resistance mechanisms remain to be explored.

Olorofim is a novel oral antifungal that targets dihydroorotate dehydrogenase, a key enzyme in fungal pyrimidine biosynthesis (4). Both prior reports and our data have demonstrated its potent *in vitro* activity against a broad spectrum of *Aspergillus* species, including azole-resistant *A. fumigatus*, *A. flavus*, and section *Nigri*, supporting its efficacy in the treatment of azole-resistant aspergillosis (43). However, of concern, the agricultural fungicide ipflufenoquin, which shares a similar mechanism of action, has been shown to induce cross-resistance to olorofim in *A. fumigatus* (44). As olorofim is an effective and well-tolerated option for patients with limited treatment choices (45), regulatory measures on agricultural ipflufenoquin use should be proposed based on field studies assessing the risk of cross-resistance and identifying high-risk agricultural practices before its widespread use to protect olorofim’s clinical efficacy.

Rezafungin is a once-weekly intravenous echinocandin that inhibits 1,3-β-D-glucan synthase (4). Ibrexafungerp is an oral triterpenoid that targets a unique site on β−1,3-D-glucan synthase, distinct from the echinocandin-binding domain (4). Our findings demonstrate *in vitro* activity of both agents against azole-resistant *A. fumigatus* and *A. flavus*, supporting their potential role in the treatment of azole-resistant aspergillosis; however, this conclusion is limited by the lack of ibrexafungerp testing against other *Aspergillus* species. Notably, whether differences between the ibrexafungerp- and echinocandin-binding sites result in limited cross-resistance between the two drug classes remains unclear, given the rarity of echinocandin-resistant isolates. Nevertheless, limited data indicate that ibrexafungerp retains activity against echinocandin-resistant *Candida* species and caspofungin-resistant *A. fumigatus* (46, 47). Its potential utility in these settings warrants further investigation through the inclusion of additional echinocandin-resistant isolates.

Fosmanogepix is an N-phosphonooxymethyl prodrug, administered orally or intravenously, that is rapidly converted *in vivo* to its active moiety, manogepix. Manogepix inhibits the fungal enzyme Gwt1, blocking GPI anchor biosynthesis, disrupting cell wall protein anchoring, and thereby compromising cell wall integrity and viability (48). Manogepix has demonstrated potent activity against diverse *Aspergillus* species in our study and prior reports (49). Its distinct mechanism of action and minimal cross-resistance with existing antifungal classes underscore its promising potential for treating drug-resistant fungal infections (50).

In comparison, both the TSARM 2016−2020 data and the present study showed consistent species-specific patterns in sample-type distribution and intrinsic or acquired resistance, as well as stable voriconazole resistance rates among *A. fumigatus* and *A. flavus* isolates. Sections *Restricti* and *Aspergillus*, reported previously, were not detected in the current surveillance, whereas new findings were identified, including azole resistance mutations (e.g., TR_46_/Y121F/T289A in *A. fumigatus* and P214L in *A. flavus*), *A. hiratsukae* (section *Fumigati*), and *A. stellatus* and *A. amoenus* (section *Nidulantes*). Notably, the proportions of itraconazole- and isavuconazole-non-WT *A. flavus* isolates were higher in the current study (4.8% and 10.3%, respectively), primarily due to an increased number of isolates with MICs one dilution above the ECVs (Table S4), highlighting the need for continued monitoring of resistance trends.

In conclusion, most clinical *Aspergillus* isolates remained susceptible to conventional antifungals, although the *A. niger* clade showed intrinsic reduced susceptibility to itraconazole. A novel P214L mutation was identified in an azole-resistant *A. flavus*, while azole-resistant *A. fumigatus* harboring environmental resistance mutations and emerging genotypes remains a concern. Novel antifungals showed broad activity, except for opelconazole, which was less effective against *A. flavus*, the *A. niger* clade, and *A. sydowii*. These findings highlight the importance of species-level identification and susceptibility testing to guide treatment, and of continued surveillance to monitor epidemiological trends and facilitate antifungal stewardship in both clinical and environmental settings in Taiwan.

## Data Availability

All data supporting the findings of this study are available within the paper and its supplemental material.

## SUPPLEMENTAL MATERIAL

Supplemental material spectrum.02659-25-s0001.docx Tables S1 to S4; Fig. S1 and S2.

ASM does not own the copyrights to Supplemental Material that may be linked to, or accessed through, an article. The authors have granted ASM a non-exclusive, world-wide license to publish the Supplemental Material files. Please contact the corresponding author directly for reuse.
