# Supplementary material for: Multicenter surveillance of antifungal susceptibility of clinical Aspergillus isolates to conventional and novel antifungal agents in Taiwan, 2021–2023
Source: Microbiol Spectr. 2026 Apr 21;14(6):e02659-25. doi: 10.1128/spectrum.02659-25 (PMC13228070; doi:10.1128/spectrum.02659-25)
Supplement: Supplemental material — Tables S1 to S4; Fig. S1 and S2. [file spectrum.02659-25-s0001.docx]

**Supplementary materials**

**Table S1.** The references for oversea azole-resistant *Aspergillus fumigatus* strains included in the microsatellite-based phylogenetic analysis

| Strain no. | Cyp51A mutation | Country | Reference |
| --- | --- | --- | --- |
| 04-202165 | TR34/L98H | Australia | (1) |
| 12-90032258 | TR34/L98H | Australia | (1) |
| 20643.017 | TR34/L98H/S297T/F495I | China | (2) |
| 20643.023 | TR34/L98H/S297T/F495I | China | (2) |
| 20677.079 | TR34/L98H/S297T/F495I | China | (2) |
| 20684.007 | TR34/L98H/S297T/F495I | China | (2) |
| C94 | TR34/L98H | China | (2) |
| C96 | TR34/L98H/S297T/F495I | China | (2) |
| C195 | TR46/Y121F/T289A | China | (2) |
| C485 | TR34/L98H/S297T/F495I | China | (2) |
| E1001 | TR34/L98H/S297T/F495I | China | (2) |
| R2-07-1_R | TR34/L98H/S297T/F495I | Denmark | (3) |
| Case 1-7d | TR34/L98H/S297T/F495I | Denmark | (4) |
| Case 2-90d | TR34/L98H | Denmark | (4) |
| Case 3-6d | TR34/L98H | Denmark | (4) |
| Case 4-36d | TR46/Y121F/T289A | Denmark | (4) |
| 14-148-2460 | TR46/Y121F/T289A | France | (5) |
| 2087 m1341.17-06-2012 | TR34/L98H | Germany | (6) |
| 2091 m1428.01-07-2012 | TR34/L98H | Germany | (6) |
| 2107 m1974.23-09-2012 | TR46/Y121F/T289A | Germany | (6) |
| A12519 | TR46/Y121F/T289A | Germany | (6) |
| 1042/09 | TR34/L98H | India | (7) |
| VPCI651/Ei/12/2/a/3 | TR46/Y121F/T289A | India | (6) |
| IFM 63432 | TR46/Y121F/T289A | Japan | (8) |
| IFM 69001 | TR34/L98H | Japan | (8) |
| IFM 69646 | TR46/Y121F/T289A | Japan | (8) |
| IFM 69694 | TR34/L98H | Japan | (8) |
| IFM 69983 | TR34/L98H | Japan | (8) |
| IFM 64460 | TR34/L98H | Japan | (8) |
| KUFF250 | TR34/L98H | Japan | (8) |
| IFM 64732 | TR34/L98H | Japan | (8) |
| E454 | TR34/L98H | Kuwait | (9) |
| 2005-456307L | TR34/L98H | The Netherlands | (10) |
| The Netherlands | TR46/Y121F/T289A | The Netherlands | (11) |
| MYC-2008-002/42 | TR34/L98H | The Netherlands | (6) |
| 094411/7/50 | TR34/L98H | The Netherlands | (6) |
| CF/NL0645 | TR46/Y121F/T289A | The Netherlands | (6) |
| CF/NL0682 | TR46/Y121F/T289A | The Netherlands | (6) |
| CF/NL2992 | TR46/Y121F/T289A | The Netherlands | (6) |
| E2619 | TR34/L98H | The Netherlands | (2) |
| F2126 | TR34/L98H/S297T/F495I | The Netherlands | (2) |
| The Netherlands 2 | TR46/Y121F/T289A | The Netherlands | (12) |
| The Netherlands 3 | TR46/Y121F/T289A | The Netherlands | (12) |
| The Netherlands 7 | TR46/Y121F/T289A | The Netherlands | (12) |
| 2015-SD202A1 | TR34/L98H/S297T/F495I | Taiwan | (13) |
| 2018-CGK-E173 | TR46/Y121F/T289A | Taiwan | (14) |
| 2012-B44 | TR34/L98H/S297T/F495I | Taiwan | (15) |
| 2013-A31 | TR34/L98H | Taiwan | (15) |
| 2014-D007 | TR34/L98H/S297T/F495I | Taiwan | (15) |
| 2014-PE10A1 | TR34/L98H/S297T/F495I | Taiwan | (15) |
| 2014-SD090A1 | TR34/L98H/S297T/F495I | Taiwan | (15) |
| 2015-E071 | TR34/L98H/S297T/F495I | Taiwan | (15) |
| 2015-HL102-1 | TR34/L98H | Taiwan | (15) |
| 2015-HL148-2 | TR34/L98H/S297T/F495I | Taiwan | (15) |
| 2015-SD202A3 | TR34/L98H | Taiwan | (15) |
| 2016-AN0106-6 | TR34/L98H/S297T/F495I | Taiwan | (15) |
| 2016-C03-004 | TR34/L98H | Taiwan | (15) |
| 2016-S05-122 | TR34/L98H | Taiwan | (15) |
| 2016-S07-008 | TR34/L98H | Taiwan | (15) |
| 2016-S14B1C3 | TR34/L98H | Taiwan | (15) |
| 2016-S16B1-1 | TR34/L98H | Taiwan | (15) |
| 2016-TC244-2 | TR34/L98H/S297T/F495I | Taiwan | (15) |
| 2017-S05-205 | TR34/L98H | Taiwan | (15) |
| 2018-S05-319 | TR34/L98H/S297T/F495I | Taiwan | (15) |
| 2018-S05-322 | TR46/Y121F/T289A | Taiwan | (15) |
| 2018-C01-009 | TR34/L98H/S297T/F495I | Taiwan | (16) |
| 2018-C03-029 | TR34/L98H | Taiwan | (16) |
| 2018-S03-006 | TR34/L98H/S297T/F495I | Taiwan | (16) |
| 2020-C01-005 | TR34/L98H/S297T/F495I | Taiwan | (16) |
| 2020-C01-015 | TR34/L98H/S297T/F495I | Taiwan | (16) |
| Tanzania | TR46/Y121F/T289A | Tanzania | (11) |

**Table S2.** *Aspergillus* isolates in this study identified by calmodulin-based phylogenetic analysis showing 98.3%–99.42% sequence identity to the closest reference species.

| Isolate with assigned species | Reference calmodulin sequence with highest identity | |
| --- | --- | --- |
|  | GenBank accession no. | Identity (%) |
| *A. brunneoviolaceus* | HE818081.1 | 99.42 |
| *A. brunneoviolaceus* | HE818082.1 | 98.82 |
| *A. flavus* | EF661508.1 | 99.16 |
| *A. japonicus* | EU021690.1 | 98.81 |
| *A. neoniger* | MH644989.1 | 99.29 |
| *A. neoniger* | MH644989.1 | 98.3 |
| *A. neoniger* | MH644989.1 | 98.3 |
| *A. neoniger* | MH644989.1 | 98.3 |
| *A. neoniger* | MH644989.1 | 98.44 |
| *A. neoniger* | LC573716.1 | 99.31 |
| *A. welwitschiae* | MH644935.1 | 99.28 |
| *A. welwitschiae* | MH644935.1 | 99.28 |
| *A. welwitschiae* | MH644935.1 | 99.28 |

**Table S3.** Distribution of *Aspergillus* sections in clinical isolates from different countries

| Country | Continent | Collection Year | Isolate No. | *Aspergillus* section (%) | | | | | Ref |
| --- | --- | --- | --- | --- | --- | --- | --- | --- | --- |
|  |  |  |  | *Fumigati* | *Flavi* | *Nigri* | *Terri* | others |  |
| Spain | Europe | 2010-2011 | 277 | 56.4 | 9.7 | 15.5 | 9.4 | 9 | (17) |
| Spain | Europe | 2014-2018 | 273 | 57.9 | 12.8 | 1.5 | 21.6 | 6.2 | (18) |
| Portugal | Europe | 2017-2018 | 156 | 44.9 | 7.1 | 25.6 | 8.3 | 14.1 | (19) |
| France | Europe | 2014-2016 | 1379 | 54.2 | 17.3 | 15.4 | 7.2 | 5.9 | (20) |
| Italy | Europe | 2016-2018 | 425 | 67.75 | 14.4 | 9.9 | 6.8 | 1.5 | (21) |
| Canada | North-America | 2018 | 113 | 76.1 | 10.6 | 4.4 | 0 | 8.9 | (22) |
| Mexico | South-America | 2006-2019 | 146 | 32.2 | 25.3 | 22.6 | 13 | 6.9 | (23) |
| Brasil | South-America | 1998-2014 | 228 | 74.6 | 13.2 | 4.4 | 4.8 | 3.1 | (24) |
| Iran | Mid-Asia | 2018-2021 | 233 | 9 | 50.2 | 33.1 | 6 | 1.7 | (25) |
| India | South-Asia | 2000-2004 | 456 | 37.7 | 46.9 | 15.4 | 0 | 0 | (26) |
| Thailand | Southeast-Asia | 2011-2019 | 79 | 48.1 | 27.9 | 16.4 | 5.1 | 2.5 | (27) |
| Indonesia | Southeast-Asia | 2012-2015 | 45 | 20 | 73.4 | 4.4 | 0 | 2.2 | (28) |
| China | East-Asia | 2011-2019 | 34 | 58.8 | 26.5 | 11.8 | 2.9 | 0 | (27) |
| China | East-Asia | 2017-2022 | 474 | 75.3 | 8.9 | 9.9 | 5.9 | 0 | (29) |
| China | East-Asia | 2018-2022 | 253 | 57.7 | 10.7 | 26.9 | 4 | 0.8 | (30) |
| Taiwan | East-Asia | 2021-2023 | 550 | 28.6 | 28.7 | 21.6 | 15.8 | 5.3 | This study |
| Japan | East-Asia | 2013-2018 | 107 | 62.6 | 6.5 | 19.7 | 5.6 | 5.6 | (31) |
| Japan | East-Asia | 2017-2018 | 130 | 43.1 | 5.4 | 37.7 | 3.1 | 10.7 | (32) |
| Australia | Oceania | 2011-2019 | 164 | 69.5 | 3 | 15.9 | 3.7 | 7.9 | (27) |

**Table S4.** Comparison of non-wild-type rates among clinical *Aspergillus* isolates and voriconazole resistance rates of *A. fumigatus* isolates between 2016–2020 and 2021–2023

|  | 2016-2020 | | |  | 2021-2023 | | | *P* value |
| --- | --- | --- | --- | --- | --- | --- | --- | --- |
| Agent/Species | No. tested | Non-WT no. (%) | 95% CI ^a^ |  | No. tested | Non-WT no. (%) | 95% CI ^a^ |  |
| Amphotericin B |  |  |  |  |  |  |  |  |
| *A. fumigatus* | 118 | 0 (0) | 0.0% – 3.1% |  | 156 | 0 (0) | 0.0% – 2.3% | 1.000 |
| *A. flavus* | 158 | 0 (0) | 0.0% – 2.3% |  | 145 | 0 (0) | 0.0% – 2.5% | 1.000 |
| *A. terreus* | 49 | 0 (0) | 0.0% – 7.3% |  | 87 | 0 (0) | 0.0% – 4.2% | 1.000 |
| *A. welwitschiae* ^b^ | 77 | 0 (0) | 0.0% – 4.7% |  | 63 | 0 (0) | 0.0% – 5.7% | 1.000 |
| *A. niger* | 16 | 0 (0) | 0.0% – 20.6% |  | 15 | 0 (0) | 0.0% – 21.8% | 1.000 |
| Itraconazole |  |  |  |  |  |  |  |  |
| *A. fumigatus* | 118 | 7 (5.9) | 2.4% – 11.8% |  | 156 | 11 (7.1) | 3.6% – 12.3% | 0.808 |
| *A. flavus* | 158 | 0 (0) | 0.0% – 2.3% |  | 145 | 7 (4.8) | 2.0% – 9.7% | **0.005** |
| *A. terreus* | 49 | 0 (0) | 0.0% – 7.3% |  | 87 | 0 (0) | 0.0% – 4.2% | 1.000 |
| *A. welwitschiae* ^b^ | 77 | 17 (22.1) | 13.4% – 33.0% |  | 63 | 17 (27.0) | 16.6% – 39.7% | 0.555 |
| *A. niger* | 16 | 2 (12.5) | 1.6% – 38.4% |  | 15 | 7 (46.7) | 21.3% – 73.4% | 0.054 |
| Voriconazole |  |  |  |  |  |  |  |  |
| *A. fumigatus* | 118 | 5 (4.2) | 1.4% – 9.6% |  | 156 | 9 (5.8) | 2.7% – 10.7% | 0.783 |
| *A. flavus* | 158 | 3 (1.9) | 0.4% – 5.5% |  | 145 | 3 (2.1) | 0.4% – 5.9% | 1.000 |
| *A. terreus* | 49 | 0 (0) | 0.0% – 7.3% |  | 87 | 0 (0) | 0.0% – 4.2% | 1.000 |
| *A. welwitschiae* ^b^ | 77 | 0 (0) | 0.0% – 4.7% |  | 63 | 0 (0) | 0.0% – 5.7% | 1.000 |
| *A. niger* | 16 | 0 (0) | 0.0% – 20.6% |  | 15 | 0 (0) | 0.0% – 21.8% | 1.000 |
| Posaconazole |  |  |  |  |  |  |  |  |
| *A. fumigatus* | 118 | 0 (0) | 0.0% – 3.1% |  | 156 | 0 (0) | 0.0% – 2.3% | 1.000 |
| *A. flavus* | 158 | 0 (0) | 0.0% – 2.3% |  | 145 | 1 (0.7) | 0.02% – 3.8% | 0.479 |
| *A. terreus* | 49 | 0 (0) | 0.0% – 7.3% |  | 87 | 0 (0) | 0.0% – 4.2% | 1.000 |
| *A. welwitschiae* ^b^ | 77 | 0 (0) | 0.0% – 4.7% |  | 63 | 0 (0) | 0.0% – 5.7% | 1.000 |
| *A. niger* | 16 | 0 (0) | 0.0% – 20.6% |  | 15 | 0 (0) | 0.0% – 21.8% | 1.000 |
| Isavuconazole |  |  |  |  |  |  |  |  |
| *A. fumigatus* | 118 | 7 (5.9) | 2.4% – 11.8% |  | 156 | 11 (7.1) | 3.6% – 12.3% | 0.808 |
| *A. flavus* | 158 | 3 (1.9) | 0.4% – 5.5% |  | 145 | 15 (10.3) | 5.9% – 16.5% | **0.003** |
| *A. terreus* | 49 | 0 (0) | 0.0% – 7.3% |  | 87 | 0 (0) | 0.0% – 4.2% | 1.000 |
| *A. welwitschiae* | 77 | 0 (0) | 0.0% – 4.7% |  | 63 | 0 (0) | 0.0% – 5.7% | 1.000 |
| *A. niger* | 16 | 0 (0) | 0.0% – 20.6% |  | 15 | 0 (0) | 0.0% – 21.8% | 1.000 |

^a^ Exact binomial 95% confidence intervals were computed using SPSS (IBM SPSS Statistics, version 25) and differences (*P* value) between the two study periods were assessed using Fisher’s exact test.

^b^ For *A. welwitschiae*, the CLSI epidemiological cutoff value for *A. niger* was applied.

**Fig. S1.** Geometric mean MICs (μg/mL) of six antifungal agents against major *Aspergillus* species (only a subset of isolates were tested for opelconazole)


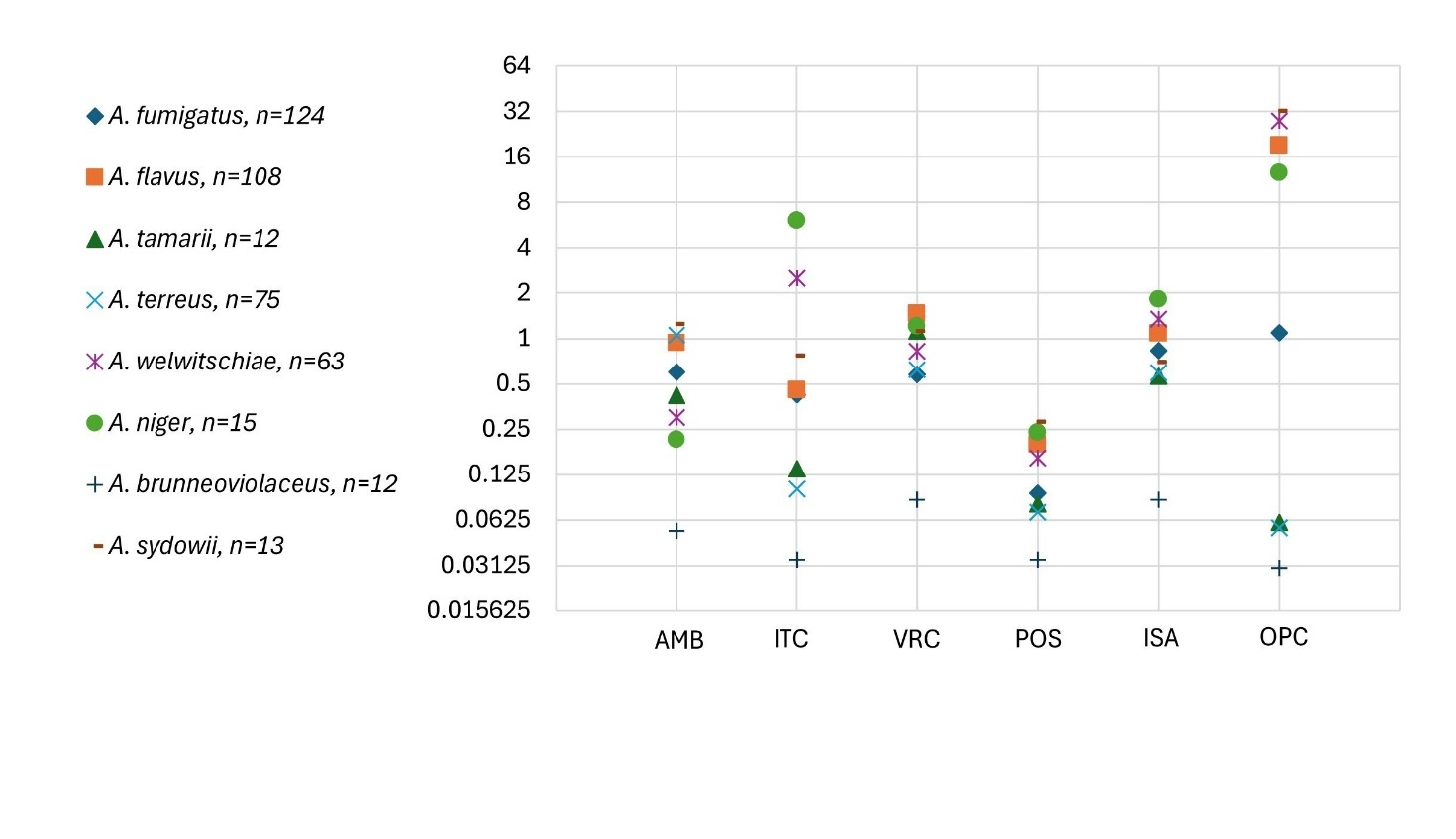


**Fig. S2.** Distribution of *Aspergillus* sections in clinical isolates from different countries (please Table S3 for details)


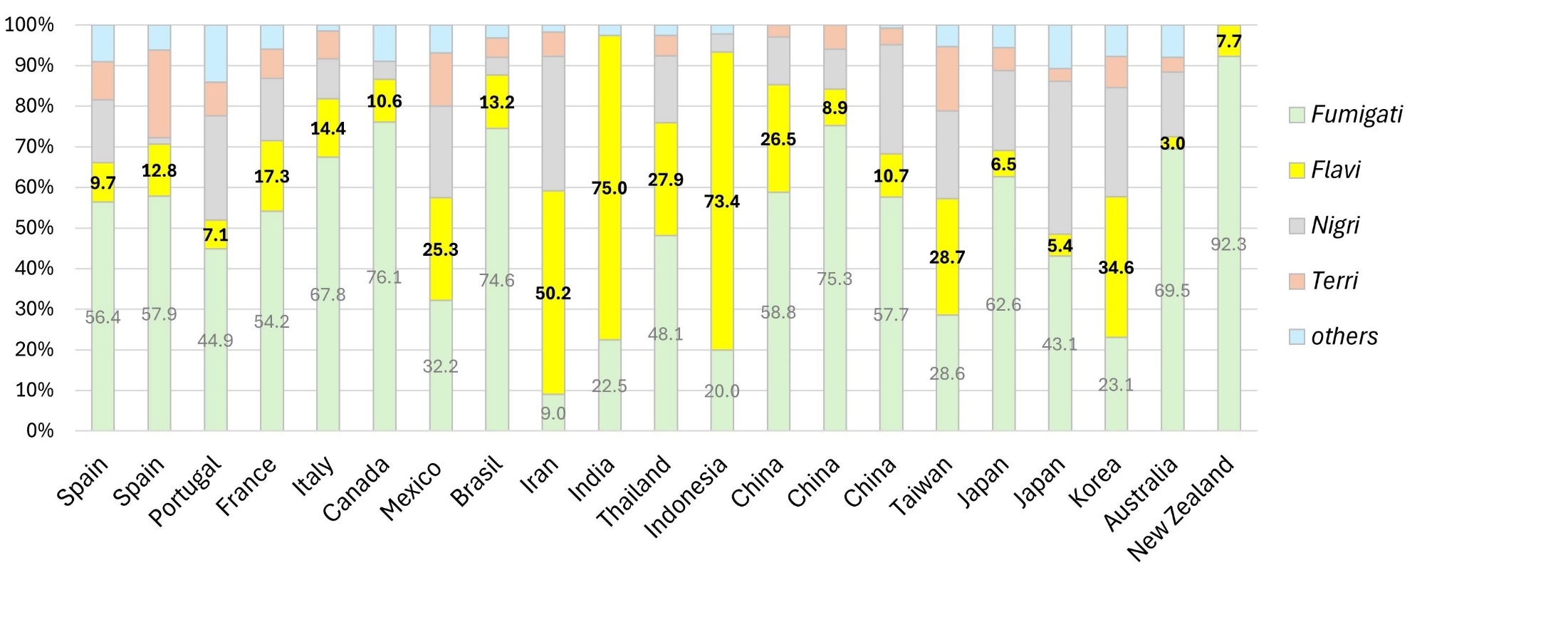


**References for the Supplementary file**

1. Kidd SE, Goeman E, Meis JF, Slavin MA, Verweij PE. 2015. Multi-triazole-resistant *Aspergillus fumigatus* infections in Australia. Mycoses 58:350-5.

2. Chen Y, Lu Z, Zhao J, Zou Z, Gong Y, Qu F, Bao Z, Qiu G, Song M, Zhang Q, Liu L, Hu M, Han X, Tian S, Zhao J, Chen F, Zhang C, Sun Y, Verweij PE, Huang L, Han L. 2016. Epidemiology and molecular characterizations of azole resistance in clinical and rnvironmental *Aspergillus fumigatus* isolates from China. Antimicrob Agents Chemother 60:5878-84.

3. Mortensen KL, Jensen RH, Johansen HK, Skov M, Pressler T, Howard SJ, Leatherbarrow H, Mellado E, Arendrup MC. 2011. *Aspergillus* species and other molds in respiratory samples from patients with cystic fibrosis: a laboratory-based study with focus on *Aspergillus fumigatus* azole resistance. J Clin Microbiol 49:2243-51.

4. Astvad KM, Jensen RH, Hassan TM, Mathiasen EG, Thomsen GM, Pedersen UG, Christensen M, Hilberg O, Arendrup MC. 2014. First detection of TR_46_/Y121F/T289A and TR_34_/L98H alterations in *Aspergillus fumigatus* isolates from azole-naive patients in Denmark despite negative findings in the environment. Antimicrob Agents Chemother 58:5096-101.

5. Lavergne RA, Morio F, Favennec L, Dominique S, Meis JF, Gargala G, Verweij PE, Le Pape P. 2015. First description of azole-resistant *Aspergillus fumigatus* due to TR_46_/Y121F/T289A mutation in France. Antimicrob Agents Chemother 59:4331-5.

6. Steinmann J, Hamprecht A, Vehreschild MJ, Cornely OA, Buchheidt D, Spiess B, Koldehoff M, Buer J, Meis JF, Rath PM. 2015. Emergence of azole-resistant invasive aspergillosis in HSCT recipients in Germany. J Antimicrob Chemother 70:1522-6.

7. Chowdhary A, Kathuria S, Xu J, Sharma C, Sundar G, Singh PK, Gaur SN, Hagen F, Klaassen CH, Meis JF. 2012. Clonal expansion and emergence of environmental multiple-triazole-resistant *Aspergillus fumigatus* strains carrying the TR₃₄/L98H mutations in the cyp51A gene in India. PLoS One 7:e52871.

8. Arai T, Majima H, Maruguchi N, Ban S, Yaguchi T, Watanabe A. 2025. Report of four azole-resistant *Aspergillus fumigatus* isolates with TR_34_ or TR_46_ mutations in Japan referred to a Mycosis Reference Center for examination. Med Mycol J 66:131-137.

9. Ahmad S, Khan Z, Hagen F, Meis JF. 2014. Occurrence of triazole-resistant *Aspergillus fumigatus* with TR_34_/L98H mutations in outdoor and hospital environment in Kuwait. Environ Res 133:20-6.

10. Badali H, Vaezi A, Haghani I, Yazdanparast SA, Hedayati MT, Mousavi B, Ansari S, Hagen F, Meis JF, Chowdhary A. 2013. Environmental study of azole-resistant *Aspergillus fumigatus* with TR_34_/L98H mutations in the cyp51A gene in Iran. Mycoses 56:659-63.

11. Chowdhary A, Sharma C, van den Boom M, Yntema JB, Hagen F, Verweij PE, Meis JF. 2014. Multi-azole-resistant *Aspergillus fumigatus* in the environment in Tanzania. J Antimicrob Chemother 69:2979-83.

12. Hagiwara D, Takahashi H, Fujimoto M, Sugahara M, Misawa Y, Gonoi T, Itoyama S, Watanabe A, Kamei K. 2016. Multi-azole resistant *Aspergillus fumigatus* harboring Cyp51A TR_46_/Y121F/T289A isolated in Japan. J Infect Chemother 22:577-9.

13. Wang HC, Huang JC, Lin YH, Chen YH, Hsieh MI, Choi PC, Lo HJ, Liu WL, Hsu CS, Shih HI, Wu CJ, Chen YC. 2018. Prevalence, mechanisms and genetic relatedness of the human pathogenic fungus *Aspergillus fumigatus* exhibiting resistance to medical azoles in the environment of Taiwan. Environ Microbiol 20:270-280.

14. Chen YC, Kuo SF, Wang HC, Wu CJ, Lin YS, Li WS, Lee CH. 2019. Azole resistance in *Aspergillus* species in Southern Taiwan: An epidemiological surveillance study. Mycoses 62:1174-1181.

15. Wu CJ, Liu WL, Lai CC, Chao CM, Ko WC, Wang HC, Dai CT, Hsieh MI, Choi PC, Yang JL, Chen YC. 2020. Multicenter study of azole-resistant *Aspergillus fumigatus* clinical isolates, Taiwan. Emerg Infect Dis 26:804-806.

16. Wang HC, Hsieh MI, Choi PC, Wu WL, Wu CJ, TSARM-Hospitals. 2023. Species distribution and antifungal susceptibility of clinical *Aspergillus* isolates: A multicentre study in Taiwan, 2016-2020. Mycoses 66:711-722.

17. Alastruey-Izquierdo A, Mellado E, Peláez T, Pemán J, Zapico S, Alvarez M, Rodríguez-Tudela JL, Cuenca-Estrella M. 2013. Population-based survey of filamentous fungi and antifungal resistance in Spain (FILPOP Study). Antimicrob Agents Chemother 57:4604.

18. Rivero-Menendez O, Soto-Debran JC, Medina N, Lucio J, Mellado E, Alastruey-Izquierdo A. 2019. Molecular identification, antifungal susceptibility testing, and mechanisms of azole resistance in *Aspergillus* species received within a surveillance program on antifungal resistance in Spain. Antimicrob Agents Chemother 63:e00865-19.

19. Sabino R, Gonçalves P, Martins Melo A, Simões D, Oliveira M, Francisco M, Viegas C, Carvalho D, Martins C, Ferreira T, Toscano C, Simões H, Veríssimo C. 2021. Trends on *Aspergillus* epidemiology-perspectives from a national reference laboratory surveillance program. J Fungi (Basel) 7:28.

20. Gautier M, Normand AC, Ranque S. 2016. Previously unknown species of *Aspergillus*. Clin Microbiol Infect 22:662-9.

21. Prigitano A, Esposto MC, Grancini A, Biffi A, Innocenti P, Cavanna C, Lallitto F, Mollaschi EMG, Bandettini R, Oltolini C, Passera M, De Lorenzis G, Sargolzaei M, Crespan M, Cogliati M, Tortorano AM, Romanò L. 2021. Azole resistance in *Aspergillus* isolates by different types of patients and correlation with environment - An Italian prospective multicentre study (ARiA study). Mycoses 64:528-536.

22. Cheng MP, Lawandi A, Lee TC, Dufresne PJ, Seidel D, Verweij PE, Cornely OA, Sheppard DC. 2019. Triazole antifungal susceptibility patterns among *Aspergillus* species in Québec, Canada. J Clin Microbiol 57:e00404-19.

23. Treviño-Rangel RJ, Villanueva-Lozano H, Bonifaz A, Castañón-Olivares LR, Andrade A, Becerril-García MA, Martínez-Reséndez MF, Ayala-Gaytán J, Montoya AM, González GM. 2021. Species distribution and antifungal susceptibility patterns of *Aspergillus* isolates from clinical specimens and soil samples in Mexico. Med Mycol 59:1006-1014.

24. Reichert-Lima F, Lyra L, Pontes L, Moretti ML, Pham CD, Lockhart SR, Schreiber AZ. 2018. Surveillance for azoles resistance in *Aspergillus* spp. highlights a high number of amphotericin B-resistant isolates. Mycoses 61:360-365.

25. Badiee P, Boekhout T, Zarei Mahmoudabadi A, Mohammadi R, Ayatollahi Mousavi SA, Najafzadeh MJ, Soltani J, Hashemi J, Diba K, Ghadimi-Moghadam A, Salimi-Khorashad AR, Shokohi T, Amin Shahidi M, Ghasemi F, Jafarian H. 2022. Multicenter study of susceptibility of *Aspergillus* species isolated from iranian university Hospitals to seven antifungal agents. Microbiol Spectr 10:e0253921.

26. Xess I, Mohanty S, Jain N, Banerjee U. 2004. Prevalence of *Aspergillus* species in clinical samples isolated in an Indian tertiary care hospital. Indian J Med Sci 58:513-9.

27. Pfaller MA, Carvalhaes CG, Rhomberg P, Messer SA, Castanheira M. 2021. Antifungal susceptibilities of opportunistic filamentous fungal pathogens from the Asia and Western Pacific Region: data from the SENTRY Antifungal Surveillance Program (2011-2019). J Antibiot (Tokyo) 74:519-527.

28. Rozaliyani A, Sedono R, Sjam R, Tugiran M, Adawiyah R, Setianingrum F, Jusuf A, Sungkar S, Hagen F, Meis JF, Wahyuningsih R. 2021. Molecular typing and antifungal susceptibility study of *Aspergillus* spp. in intensive care unit (ICU) patients in Indonesia. J Infect Dev Ctries 15:1014-1020.

29. Bilal H, Zhang D, Shafiq M, Khan MN, Chen C, Khan S, Cai L, Khan RU, Hu H, Zeng Y. 2023. Epidemiology and antifungal susceptibilities of clinically isolated *Aspergillus* species in South China. Epidemiol Infect 151:e184.

30. Hussain A, Wang Y, Mo E, Khan MN, Zhang Q, Li L, Zhu J, Zhu M. 2024. Epidemiology and antifungal susceptibilities of clinically isolated *Aspergillus* species in tertiary hospital of southeast China. Infect Drug Resist 17:5451-5462.

31. Toyotome T, Saito S, Koshizaki Y, Komatsu R, Matsuzawa T, Yaguchi T. 2020. Prospective survey of *Aspergillus* species isolated from clinical specimens and their antifungal susceptibility: A five-year single-center study in Japan. J Infect Chemother 26:321-323.

32. Tsuchido Y, Tanaka M, Nakano S, Yamamoto M, Matsumura Y, Nagao M. 2019. Prospective multicenter surveillance of clinically isolated *Aspergillus* species revealed azole-resistant *Aspergillus fumigatus* isolates with TR_34_/L98H mutation in the Kyoto and Shiga regions of Japan. Med Mycol 57:997-1003.
